# Professional Ethics

**Published:** 1894-07

**Authors:** 


### Professional Ethics.

Prior to the graduation of the class of the College of Dental Surgery, of the University of Michigan the following article was prepared and signed by all of the members of the graduating class. It consists simply in an agreement or promise to abide by the requirements of the code of ethics of the American Dental Association and of all other dental association in affiliation with that body. Such an agreement exercises a wholesome influence upon all who promise to conform to its requirements, it also, in a good measure, affords a degree of moral support and strength to everyone, and constitutes a bond of union that should not be lightly esteemed. It is as follows:

"To maintain its dignity and to further the honor of the profession which I am about entering, and in loyalty to my Alma Mater and the desire and precepts of its faculty. I the undersigned do willingly and cheerfully promise to abide by the code of ethic of the American Dental Association, and will strive to deport myself in such manner as to claim the respect of the public, the good will of my co-workors in the profession, and the continued esteem of my instructors and brother graduates. If at any time, I should fail to so act, I shall expect to forfeit all claims to personal or professional recognition by my Alma Mater and classmates."

